# Supplementary material for: Explore barriers to using the internet for health information access in African countries: A systematic review
Source: PLOS Digit Health. 2025 Jan 27;4(1):e0000719. doi: 10.1371/journal.pdig.0000719 (PMC11771857; doi:10.1371/journal.pdig.0000719)
Supplement: Attachment — Submitted filename: Point by point.docx [file pdig.0000719.s003.docx]

**Dear Editors,**

We would like to express our sincere gratitude to the editorial team at PLOS Digital Health for the opportunity to revise and resubmit the manuscript titled "Explore Barriers to Using the Internet for Health Information Access in African Countries: A Systematic Review." We greatly appreciate the constructive feedback and thoughtful suggestions provided during this second revision. Your professionalism and dedication to maintaining high publication standards have been invaluable throughout the process.

Thank you again for your continued support and guidance.

Best regards,

*** Corresponding author:**

**Alex Ayenew Chereka**

**Email:** [zemeneayenew@gmail.com](mailto:zemeneayenew@gmail.com)

**Point by point response for the reviewers**

**Dear Reviewer 2,**

We would like to express our sincere and heartfelt gratitude for the time and expertise you have dedicated to reviewing our manuscript. You’re thoughtful and thorough feedback during this second revision has been invaluable in guiding us to improve the quality and clarity of our work. We deeply appreciate the constructive suggestions you provided, which have significantly enhanced both the depth and rigor of our research.

Your detailed comments have helped us address key areas for improvement, and your insightful recommendations have been crucial in refining our arguments and analysis. We are particularly grateful for the careful consideration you gave to the revisions, which has undoubtedly strengthened the manuscript overall.

**Reviewer #2:** I would like to appreciate the authors for the detailed response to my initial feedback. I particularly acknowledge their response as to why the older articles should and were included in the review. Also, their effort to widen the scope of the search to include more articles and the limitation to doing this are also acknowledged.

However, I believe the analysis of the findings and discussion is still not robust enough even based on the fewer number of articles reviewed. There are also inconsistencies in their feedback and inferences.

**Some of my concerns are listed below**

**Comment 1:**While Table one provided the characteristics of the 6 articles that met the inclusion criteria, Table 2 which describes the frequency of the barriers mentioned in the articles, made mention of 8 articles with reference numbers 20, 22, 25, 27, 28, 37, 38 and 39. Even though 20 and 39 are the same articles; 27 in a distinct article, thus making the articles 7. This is a red flag for me.

**Authors answer:** Thank you for your careful review and valuable feedback. We appreciate your attention to detail regarding the discrepancy between Table 1 and Table 2. Upon revisiting the manuscript, we confirmed that references 20 and 39 are indeed the same article, and we have revised the document to ensure that this reference is properly cited only once. Additionally, reference number 27 was mistakenly included, as the full text of this article was not available at the time of our analysis.

To address these issues, we have removed the redundant citation of reference 39 and excluded reference number 27 entirely. This revision ensures the accuracy and consistency of the references across both tables. We sincerely appreciate your constructive comment, which has helped us improve the clarity and precision of our work.

**Comment 2:** The authors also mention that they “revisited our analysis with your comments in mind to explore potential differences between the challenges faced by students and health workers. While we agree that these groups might encounter different challenges, our analysis did not reveal significant differences in the context of the studies reviewed”. But looking at the data presented in Table 2, the study with reference 38 which was on secondary school students in Nigeria reported 8 out of the 11 barriers while college students in Ghana (37) reported 10 out of the 11 barriers while the study on (20 and 39) reported three barriers. This sorts of show clear distinction in the magnitude and even types of barriers experienced by the different categories, and I expect the authors to have looked more into these findings and provide possible insights into factors that might have been responsible for the findings e.g. policy environment, socio-economic factors, Location etc.

**Authors answer:** Thank you for this insightful comment. We appreciate the detailed observation regarding the variations in barriers reported across different groups, as presented in Table 2. Specifically, secondary school students in Nigeria (ref. 38) reported 8 out of 11 barriers; college students in Ghana (ref. 37) reported 10, whereas health professionals in Nigeria (ref. 20) reported only 3 barriers. The barriers reported by health professionals (ref. 20) were slow internet connection, cost of internet access, lack of information-searching skills, and too much information. Notably, "too much information" was a factor for health professionals but not for the other groups.

These observed differences likely reflect variations in policy environments, socio-economic contexts, and infrastructure quality across the respective settings. For instance, students may encounter higher costs, limited access to technology, and less institutional support compared to health professionals, who may have better resources in their work environment. However, health professionals may face different challenges, such as information overload, which may be less relevant to students due to their differing needs.

**Comment 3:** Also, in their response to the need to widen the scope of the search, the authors “observed that while the titles of the newly identified articles were similar to those already included, their results and contents differed significantly. Due to these differences, we have opted to retain the original set of articles in order to maintain consistency and relevance to our research objectives”. This sort of implies some form of bias in the application of the inclusion criteria.
**Authors answer:** We appreciate the reviewer’s concern regarding potential bias in our inclusion criteria. To clarify, although the newly discovered articles had similar titles to those already included in our review, we found that their results and content were substantially different. These differences included variations in study design, populations, methodologies, and the specific research questions addressed, which were not aligned with the focus of our review.

As a result, we decided to retain only the original set of articles to maintain consistency and relevance to our research objectives. We acknowledge that this decision may introduce some selection bias by excluding studies with potentially relevant findings. However, we believe this approach was necessary to ensure the homogeneity of the included studies.

**Comment 4:** I am also curious about the barrier “low frequent use of the internet” as a distinct barrier. This sound like a circular reference since almost all the other barriers could lead to infrequent use of the internet.

**Authors answer:** Thank you for your insightful observation regarding the barrier “low frequent use of the internet.” We understand that this could appear as a circular reference, as other barriers may indeed contribute to reduced internet use.

To clarify, we included "low frequent use of the internet" as a distinct barrier because it was specifically mentioned as an independent factor in several studies, with unique implications for digital access and engagement. Although it is influenced by other barriers, it also stands on its own as a behavioral pattern that limits digital interaction, regardless of the underlying causes. This pattern can have independent effects on skill development, confidence, and willingness to use online resources, and thus, we felt it warranted separate consideration. We appreciate your feedback and are happy to provide further clarification if needed.

**Comment 5:** There are other observations in the discussions and even in the background section. I thus believe that more works needs to be done on the article to meet the rigor expected of an article to be published in PLOS.

**Authors answer:** Thank you for your valuable feedback. We appreciate your observations regarding the discussions and the background section. In response to your comments, we have carefully revised the manuscript and addressed the points raised, ensuring that the rigor expected of an article to be published in PLOS is met. The necessary revisions have been incorporated, and we believe that the manuscript now reflects the required depth and clarity. Please find the updated version attached for your review. We hope the changes meet your expectations and look forward to your further guidance.
