# Supplementary material for: Explore barriers to using the internet for health information access in African countries: A systematic review
Source: PLOS Digit Health. 2025 Jan 27;4(1):e0000719. doi: 10.1371/journal.pdig.0000719 (PMC11771857; doi:10.1371/journal.pdig.0000719)
Supplement: Attachment — Submitted filename: Point-by-point-response.docx [file pdig.0000719.s002.docx]

**Dear Academic Editor of PLOS Digital Health**

We hope this message finds you well. We are writing to express our heartfelt gratitude for your invaluable support and guidance throughout the editorial process of our manuscript. We are pleased to submit the revised version of our manuscript, titled “***Explore Barriers to Using the Internet for Health Information Access in African Countries: A Systematic Review” (PDIG-D-24-00233).***

We sincerely appreciate the insightful comments and constructive feedback provided by you and the reviewers. Your dedication to excellence has been crucial in enhancing the clarity, rigor, and overall quality of our research. Your contributions have been instrumental in addressing key areas and significantly improving our manuscript.

Thank you for considering our revised submission. We eagerly await your feedback and remain available for any further revisions or clarifications.

Best regards,

**Corresponding Author**:

**Alex Ayenew Chereka**

**Email:** [zemeneayenew@gmail.com](mailto:zemeneayenew@gmail.com)

# 1. Point-by-point response letter to Editor

**Dear Editor,**

Once again, we greatly appreciate the time and expertise you dedicated to providing thorough and thoughtful feedback. We provide a point-by-point response addressing the changes based on your suggestions and comments as follows:

**Editor**: Please submit your revised manuscript within 30 days. Please include the rebuttal letter for the response of the reviewer and editor, marked, and unmarked document when submitting your revised manuscript.

**Authors:** We sincerely appreciate your insightful comment. We have taken your feedback seriously and diligently addressed the concern you mentioned. Additionally, we have ensured that all necessary documents, as per your recommendation, have been submitted along with the revised manuscript within a given time. Thank you for guiding us through this process.

**Editor comment-1:** ournal Requirements:

1. In the online submission form, you indicated that "All the data used for the current study are available from the corresponding author upon reasonable request.".

 All PLOS journals now require all data underlying the findings described in their manuscript to be freely available to other researchers, either

a. In a public repository,

b. Within the manuscript itself, or

c. Uploaded as supplementary information..

**Authors**: Thank you for highlighting this issue. We sincerely apologize for any confusion caused. In accordance with PLOS journal requirements, we have revised the statement regarding data availability. The updated statement now reads: "All the data used for the current study are available Within the manuscript itself."

**Editor comment-2**: Please review your reference list to ensure that it is complete and correct. If you have cited papers that have been retracted, please include the rationale for doing so in the manuscript text, or remove these references and replace them with relevant current references. Any changes to the reference list should be mentioned in the rebuttal letter that accompanies your revised manuscript. If you need to cite a retracted article, indicate the article’s retracted status in the References list and also include a citation and full reference for the retraction notice.

**Authors**: Thank you for your instructions regarding the reference list. We have reviewed our manuscript, and no retracted articles were cited. Therefore, no changes were necessary in this regard. We appreciate your attention to this matter.

# 2. Point-by-point response letter to Reviewer-1

**Authors**: Thank you for your valuable feedback on our manuscript, " ***Explore barriers to using the Internet for health information access in African countries: A Systematic Review" (PDIG-D-24-00233)***." We greatly appreciate the time and effort you have invested in providing insightful comments and suggestions. We have thoroughly considered your feedback and made the necessary revisions. Below, we address each of the points raised

**Reviewer-1 comment-1:** Modify Figure 1: Ensure it accurately reflects the data and improves clarity.’

**Authors**: Thank you for your valuable feedback regarding Figure 1. We have carefully reviewed and modified the figure to ensure it accurately reflects the data and enhances clarity. The updated figure has been included in the revised manuscript.

**Reviewer-1 comment-2:** Editorial Issues: Replace "show figure" and "show Table" with (Fig. 1), (Fig. 2), or (Table 1) for consistency and professionalism.

**Authors**: Thank you for your careful review and valuable feedback. We appreciate your attention to detail regarding the editorial issues. We have revised the manuscript to replace "show figure" and "show table" with the appropriate references, such as (Fig. 1), (Fig. 2), and (Table 1), to ensure consistency and professionalism throughout the document. We kindly invite you to review the updated manuscript on **pages 8 and 9.**

**Reviewer-1 comment-3:** Results Section: Remove the search strategy details, as they are already covered in the Methods section, to avoid redundancy.’

**Authors**: Thank you for your suggestion. We agree that the search strategy details are more appropriate for the Methods section and should not be repeated in the Results section. We have revised the Results section to remove the redundant information and ensure that the focus remains on presenting the findings.

**Reviewer-1 comment-4:** Citations: Use “Ibegbulam, et al.” rather than “Ibegbulam, I.J., et al.” for better readability and standardization.

**Authors**: Thank you for your suggestion regarding the citation format. We have updated all relevant citations to use the format "Ibegbulam, et al." as recommended, to enhance readability and ensure consistency throughout the manuscript.

**Reviewer-1 comment-5:** Patient and Public Involvement: Move this section to the Methods to maintain logical structure.

**Authors**: Thank you for your insightful feedback. I agree that moving the "Patient and Public Involvement" section to the Methods would enhance the logical flow and structure of the manuscript. I have made the necessary adjustment, relocating this section to the Methods, ensuring it aligns with the overall organization of the paper. We kindly invite you to see the updated manuscript on **page 4, lines 99-105.**

**Reviewer-1 comment-6:** Avoid Abbreviations: Refrain from using abbreviations in captions, titles, or abstracts to ensure clarity for all readers.

**Authors**: Thank you for your valuable feedback. We acknowledge the importance of clarity in communicating research findings. To address your concern, we have removed all abbreviations from the captions, titles, and abstract. This change will help ensure that the content is easily understood by all readers.

**Reviewer-1 comment-7:** Recommendations: Clearly separate recommendations from the conclusion to highlight actionable insights.

**Authors**: Thank you for your insightful suggestion regarding the recommendations section. We have revised the manuscript to clearly separate the recommendations from the conclusion. This change highlights the actionable insights derived from the study and ensures that the recommendations are distinct and easily identifiable.

**Reviewer-1 comment-8:** Number Headings Chronologically: Ensure all Heading 1 sections are numbered in chronological order for better organization.

**Authors**: Thank you for your valuable feedback regarding the numbering of the headings. We have revised the manuscript to ensure that all Heading 1 sections are now numbered in chronological order, improving the overall organization and clarity of the document.

# 3. Point-by-point response letter to Reviewer-2

**Dear Reviewer,**

Thank you for your insightful feedback on our manuscript, "*Explore Barriers to Using the Internet for Health Information Access in African Countries: A Systematic Review" (PDIG-D-24-00233)*. We deeply appreciate the time and effort you dedicated to reviewing our work. We have carefully considered your comments and implemented the necessary revisions. Below, we address each of your points in detail.

**Reviewer-2 comment-1:**

This study aims to systematically review the literature to explore barriers to accessing health information on the Internet in African countries. The focus of the study is appropriate, in that it seeks to understand the barriers to the use of the internet especially in this ICT era, where an increasing number of the general public including professionals seek health information from the internet. The use of PRISMA to guide the methodology is commendable; the inclusion and exclusion criteria where clearly outlined and the selection process where transparent with multiple reviewers, which minimizes bias.

However, there are some fundamental gaps in the analysis and conclusion of the study. The inclusion of articles published in 2006 and even the two published in 2017 and 2018 in a review done and to be published in 2024 on a fast-paced issue such as the topic of this study, limits the significance and relevance of the conclusions of the study.

**Authors**: Thank you for your valuable feedback. We acknowledge your concern regarding the inclusion of articles published as early as 2006, as well as those from 2017 and 2018, in a review set to be published in 2024.

While we agree that the field is rapidly evolving and that the relevance of older studies may diminish over time, we believe the inclusion of these articles is justified for several reasons:

1. Historical Context: The older articles provide a crucial historical baseline for understanding the evolution of this topic. They help illustrate how the field has developed over time and allow us to identify trends or shifts in perspectives and findings.

2. Continued Relevance: Many of the older studies included still offer valuable insights and foundational knowledge that remain relevant despite the passage of time. We carefully assessed these studies for their ongoing significance and included only those that contribute meaningfully to our analysis.

3. Comprehensive Review: Our goal was to provide a comprehensive review that captures the full breadth of research available on this topic. Excluding older but still pertinent studies could lead to a skewed understanding of the subject matter.

4. Limitations and Sensitivity Analysis: We have acknowledged the limitations of including older studies in our analysis section. Additionally, we conducted sensitivity analyses to ensure that our conclusions remain robust, even when these older studies are excluded. Our findings indicate that the inclusion or exclusion of these articles does not significantly alter the overall conclusions of the review.

To address your concern further, we will revisit our analysis to ensure that our conclusions are both timely and relevant. We will consider giving more weight to recent studies while still acknowledging the contributions of older works.

**Reviewer-2 comment-2:**

The analysis should also have been more in-depth than what was presented. The respondents in the articles reviewed were either students (three articles) or health workers (two articles) and this should have been factored in the analysis since the challenges the two groups faced might have been different and could have influenced the conclusions. Moreso there were no articles that respondents were from other categories of the general public and this this should have been factored in the analysis, discussions and conclusions. The analysis is thus rather too simplistic - it just describes the number of journals that mentioned the different

**Authors**: Thank you for your insightful feedback. We revisited our analysis with your comments in mind to explore potential differences between the challenges faced by students and health workers. While we agree that these groups might encounter different challenges, our analysis did not reveal significant differences in the context of the studies reviewed. Therefore, we presented the findings as a collective overview. We also recognize the absence of articles that included respondents from other categories of the general public. This limitation has now been noted in the revised discussion, where we address its potential impact on the generalizability of our conclusions.

We apologize for any confusion this might have caused and appreciate your input, which has helped us clarify our approach and findings

**Reviewer-2 comment-3:**

barriers and then made policy recommendations based on that.

I would thus suggest that the authors explore further refining their search criteria to possibly get more articles. The analysis and discussion should also be more in-depth to justify the conclusions and recommendations made.

**Authors**: Thank you for your valuable feedback. In response to your suggestion, we have refined our search strategy to include additional articles. However, we observed that while the titles of the newly identified articles were similar to those already included, their results and contents differed significantly. Due to these differences, we have opted to retain the original set of articles in order to maintain consistency and relevance to our research objectives.

We have also taken steps to enhance the depth of our analysis and discussion. The revised manuscript now includes a more comprehensive examination of the barriers identified in the included studies, and we have provided a more detailed justification for our conclusions and policy recommendations. We believe these revisions address your concerns and strengthen the overall quality of the manuscript. Thank you once again for your constructive comments. See the revised manuscript on ***page 13 lines 313-328***

# 4. Point-by-point response letter to Reviewer-3

**Dear Reviewer,**

Thank you for your thoughtful and detailed feedback on our manuscript, "Explore Barriers to Using the Internet for Health Information Access in African Countries: A Systematic Review" (PDIG-D-24-00233). We sincerely appreciate the time and effort you put into reviewing our work. We have carefully reviewed your comments and made the necessary revisions. Below, we provide a detailed response to each of your observations**.**

**Reviewer-3 comment-1:**

Methods

- The authors should consider stating the study period in the methods section.
- The authors should remove the distinct headings within the Abstract.

**Authors**: Thank you for your insightful suggestions and constructive comments. We have now included the study period in the Methods section as recommended. Kindly refer to the revised manuscript on ***page 5, lines 131-133.***

Regarding the distinct headings within the Abstract, we understand your concern. However, we included these headings to align with the requirements of the journal to which we are submitting. The journal's guidelines specifically request that abstracts be structured with distinct headings for clarity and consistency.

We hope this explanation addresses your concerns. Please let us know if any further adjustments are needed..

## Reviewer-3 comment-2

Introduction

- The last three sentences in the 3^rd^ paragraph need references.
- The first sentence and the last 3 sentences in paragraph 4 need to be referenced.

**Authors**: Thank you for your detailed feedback on the introduction section. We have reviewed the third and fourth paragraphs as per your suggestion and added the necessary references to support the following statements:

In the third paragraph, we have included references for the last three sentences to substantiate the claims made.

In the fourth paragraph, we have added references for the first sentence and the last three sentences, ensuring that all statements are adequately supported by relevant literature. We appreciate your constructive input, which has strengthened the clarity and credibility of our introduction.

**Reviewer-3 comment-3**

Inclusion and exclusion criteria

Inclusion criteria

- The authors should consider introducing inclusion criteria as a subtitle under the Inclusion and exclusion criteria.
- The authors need to state the study period in the inclusion criteria.

**Authors**: Thank you for your valuable feedback. We have revised the "Inclusion and exclusion criteria" section by removing the separate "Inclusion criteria" subtitle to enhance consistency and improve the overall flow of the manuscript. As for the study period, it has already been detailed in the Methods section, where we believe it is best placed for clarity and to avoid redundancy. Kindly refer to the revised manuscript on ***page 5, lines 131-133.***
