# Supplementary material for: Explore barriers to using the internet for health information access in African countries: A systematic review
Source: PLOS Digit Health. 2025 Jan 27;4(1):e0000719. doi: 10.1371/journal.pdig.0000719 (PMC11771857; doi:10.1371/journal.pdig.0000719)
Supplement: Attachment — Submitted filename: Point by point F.docx [file pdig.0000719.s004.docx]

**Dear Editors,**

We would like to express our sincere gratitude to the editorial team at PLOS Digital Health for the opportunity to revise and resubmit the manuscript titled **"Explore Barriers to Using the Internet for Health Information Access in African Countries: A Systematic Review."** We deeply appreciate the constructive feedback and thoughtful guidance provided throughout this process. Your professionalism and dedication to upholding high publication standards have been invaluable in helping us improve the quality of our work.

We are truly grateful for your continued support and assistance as we worked to address the journal’s requirements and improve the manuscript further.

**Point-by-Point Response**

Unfortunately, we were unable to access any attached comments from the reviewers or editors for this revision. To address this issue, we contacted **Dr. Laura Sbaffi (Section Editor, *PLOS Digital Health*) and Mr. Oliver Ian Tolentino (Journal Assistant, *PLOS Digital Health*)** to request clarification or access to the reviewer and editor comments. However, we did not receive any feedback as an attachment.

In the absence of specific comments, we conducted a thorough revision of the manuscript to align it with the journal's standards. The following key changes were made:

**Abstract (Methods Section)**

The description of the methodology was refined for clarity and to ensure it accurately reflects the systematic review process.

**Methods Section (Inclusion and Exclusion Criteria)**

The criteria for study inclusion and exclusion were revised and clearly articulated to enhance transparency.

**Discussion Section**

We improved the interpretation of significant findings by elaborating on key variables and their implications in the context of the study.

**Strengths and Limitations Section**

This section was revised to provide a balanced perspective, highlighting the study's contributions and areas for improvement.

**Overall Grammar and Language Review**

The entire manuscript was carefully reviewed for grammar, clarity, and consistency to ensure the text meets the journal’s linguistic and scientific standards.

We trust that these revisions address potential concerns and align the manuscript with the journal’s requirements. If additional feedback or clarifications are needed, we are happy to make further modifications.

Thank you for your time, guidance, and support throughout this process.

**Best regards,**

On behalf of all authors

**Alex Ayenew Chereka**

*Corresponding Author*

Email: [*zemeneayenew@gmail.com*](mailto:zemeneayenew@gmail.com)
