# Supplementary material for: Explore barriers to using the internet for health information access in African countries: A systematic review
Source: PLOS Digit Health. 2025 Jan 27;4(1):e0000719. doi: 10.1371/journal.pdig.0000719 (PMC11771857; doi:10.1371/journal.pdig.0000719)
Supplement: Attachment — Submitted filename: Reviewer comments-SR- 27-07-24.docx [file pdig.0000719.s001.docx]

**Manuscript title: Explore barriers to using the internet for health information access in African countries: A Systematic Review.**

Abstract

Methods

- The authors should consider stating the study period in the methods section.
- The authors should remove the distinct headings within the Abstract.

Main text

Introduction

- The last three sentences in the 3^rd^ paragraph need references.
- The first sentence and the last 3 sentences in paragraph 4 need to be referenced.

Methods

Inclusion and exclusion criteria

Inclusion criteria

- The authors should consider introducing inclusion criteria as a subtitle under the Inclusion and exclusion criteria.
- They authors need to state the study period in the inclusion criteria.
